# Human Dendritic Cell Functional Specialization in Steady-State and Inflammation

**DOI:** 10.3389/fimmu.2014.00131

**Published:** 2014-04-01

**Authors:** Arjan Boltjes, Femke van Wijk

**Affiliations:** ^1^Laboratory for Translational Immunology, Department of Pediatric Immunology, University Medical Center Utrecht, Utrecht, Netherlands

**Keywords:** dendritic cells, subsets, monocytes, humans, inflammation, inflammatory dendritic cells, functional specialization, skin

## Abstract

Dendritic cells (DC) represent a heterogeneous population of antigen-presenting cells that are crucial in initiating and shaping immune responses. Although all DC are capable of antigen-uptake, processing, and presentation to T cells, DC subtypes differ in their origin, location, migration patterns, and specialized immunological roles. While in recent years, there have been rapid advances in understanding DC subset ontogeny, development, and function in mice, relatively little is known about the heterogeneity and functional specialization of human DC subsets, especially in tissues. In steady-state, DC progenitors deriving from the bone marrow give rise to lymphoid organ-resident DC and to migratory tissue DC that act as tissue sentinels. During inflammation additional DC and monocytes are recruited to the tissues where they are further activated and promote T helper cell subset polarization depending on the environment. In the current review, we will give an overview of the latest developments in human DC research both in steady-state and under inflammatory conditions. In this context, we review recent findings on DC subsets, DC-mediated cross-presentation, monocyte-DC relationships, inflammatory DC development, and DC-instructed T-cell polarization. Finally, we discuss the potential role of human DC in chronic inflammatory diseases.

## Introduction

Dendritic cells (DC) have highly effective mechanisms to detect and capture antigens and to subsequently determine the magnitude and quality of adaptive immune responses. They are the most potent antigen-presenting cells (APC) in promoting activation of naïve T cells.

In the past years, major advances have been made in understanding mouse DC ontogeny, molecular development, and function. Human DC immunobiology, however, is only beginning to be understood. Several recent reviews have aligned data on human and mouse DC networks (1, 2) and have highlighted similarities, parallels, and differences. In the current review, we will put the human DC subsets in the spotlight and focus on recent advances in human DC development and functional specialization, both in steady-state and in inflammatory conditions.

### Human DC subsets

**Figure 1:**
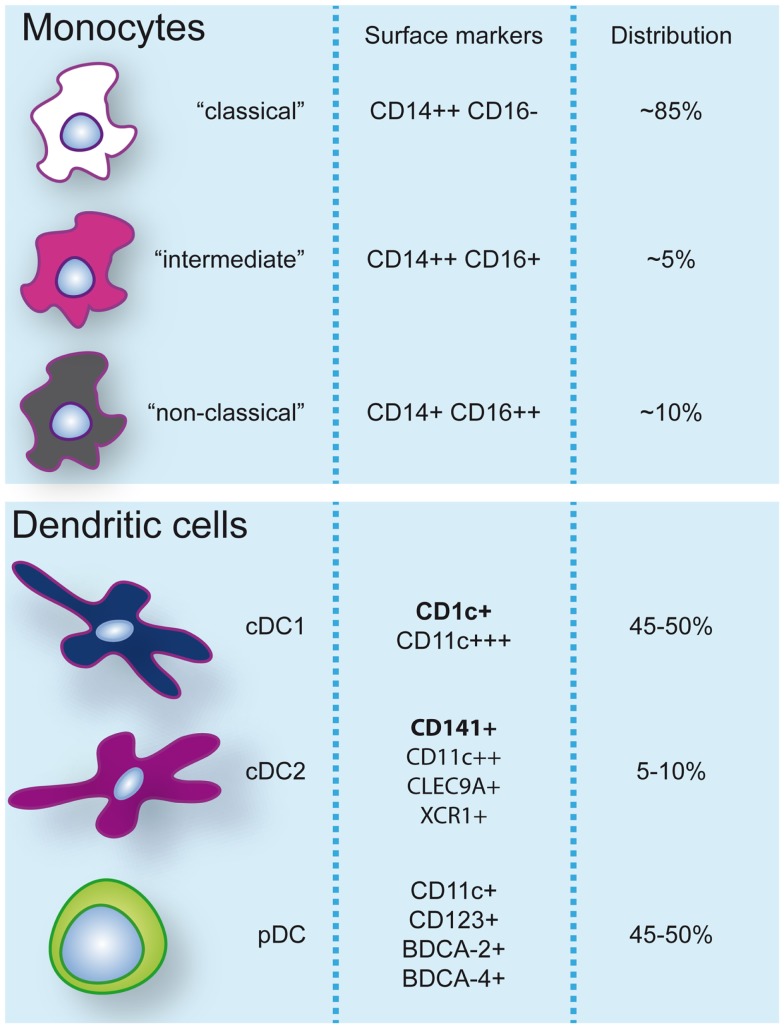
**Monocyte and dendritic cell populations in human peripheral blood during steady-state conditions**. Expression of markers commonly used to identify and discern these populations are indicated. Additionally, frequencies (%) of monocyte subsets within the monocyte pool and dendritic cells within the dendritic cell pool are specified.

Dendritic cells are heterogeneous and can be sub-classified based on anatomical location, origin, and function. After exiting the bone marrow into the blood, DC progenitors give rise to resident and migratory DC following final *in situ* differentiation. Resident DC are localized in lymphoid tissue (LT) where they take up antigen from the lymph and bloodstream and present it to local T cells. Non-lymphoid tissue (NLT) DC constitutively migrate from the tissues to the lymph nodes where they present tissue-derived antigens to T cells. In peripheral blood, three main DC subsets have been acknowledged within the HLA-DR^+^ lineage negative fraction that can be identified based on their surface marker expression: plasmacytoid DC (pDC) and two types of conventional DC (cDC); CD1c/BDCA-1^+^ cDC, and CD141/BDCA-3^+^ cDC (3, 4) (Figure 1). These subsets can also be found in spleen and tonsils (5–7). Hierarchical clustering of mouse LN and human blood DC subsets based on genome-wide expression profiling demonstrated clustering of human pDC with mouse pDC, CD141^+^ cDC with mouse CD8α^+^ DC, and human CD1c^+^ cDC with mouse CD11b^+^ DC (8). The total blood DC population consists of about 5–10% CD141^+^ cDC while the remainder is divided into equal parts pDC and CD1c^+^ cDC (9).

Segura and colleagues recently demonstrated that resident LN DC subtypes also phenotypically correspond to blood pDC, CD1c^+^, and CD141^+^ cDC (10). Whereas cDC are thought to reach the LN after trafficking from peripheral tissue via afferent lymphatic vessels, pDC have been described to migrate directly from the blood to the LN via high endothelial venules (HEV) (11).

In general, NLT DC have a more activated phenotype expressing higher levels of co-stimulatory molecules than their blood counterparts. In the skin, lung, and liver two cDC subsets identical to CD1c^+^ and CD141^+^ blood cDC have been identified (12, 13), whereas pDC are absent in tissues under steady-state conditions. Human CD1c^+^ and CD141^+^ tissue-resident DC are related to mouse tissue-resident CD11b^+^ and CD103^+^ DC, respectively (12). In addition to cDC, human tissues also harbor migratory CD14^+^ DC, which are not found in the blood (12, 13) and do not have an identified murine equivalent. However, CD14^+^ NLT DC display an intermediate phenotype expressing both macrophage and DC markers, and the transcriptomic expression profiles of skin and lung CD14^+^ DC recently were demonstrated to be most closely related to blood monocytes and tissue macrophages (12, 13), which questions their DC origin.

### DC development

In mice, the macrophage and DC progenitor (MDP) gives rise to monocytes and the common DC progenitor (CDP). The latter has lost the potential to generate monocytes and macrophages [reviewed by Ref. (14, 15)]. Recent fate-mapping and cellular barcoding studies have confirmed cDC as an independent hematopoietic lineage (16, 17). In the bone marrow, CDP give rise to pre-DC that migrate via the blood into LN and NLT, and terminally differentiate into LN and NLT cDC subsets. Once in the periphery, DC have a short life-span and retain a high degree of plasticity that enables a rapid and diverse response to specific extrinsic stimuli.

Human DC arise from BM precursors and it has recently been shown that both granulocyte-macrophage progenitors (GMP) and multi-lymphoid progenitors (MLP) can generate DC *in vitro* or following adoptive transfer experiments in mice (18). Consistent with this, *in vivo* data on human DC deficiency caused by a GATA2 mutation demonstrate that a complete absence of MLP (and only reduced numbers of GMP) results in loss of all DC (19). It is unknown however, whether committed DC progenitors exist in humans. So far, equivalents of mouse MDP, CDP, and pre-DC have not been found. Identification of early DC precursors in human blood is complicated by the fact that, in contrast to mouse DC precursors, human CD34^+^ hematopoietic stem cells already express MHC class II. It has been speculated that human circulating cDC may be regarded as pre-DC that undergo their final DC differentiation stage *in situ* (4, 10, 13). CD1c^+^ and CD141^+^ cDC proliferate more in the blood than in the LN (10, 20), suggesting that in the blood they are not fully differentiated. In contrast to cDC, both blood and LN-derived pDC do not proliferate (10, 20), supporting the idea that pDC leave the bone marrow fully differentiated (21).

## Human Mononuclear Phagocyte System: How DC Differ from Monocytes and Macrophages

The human mononuclear phagocyte system has historically been divided into macrophages, monocytes, and DC, with classification mainly based on phenotypic, anatomical, and/or functional criteria. In this classification, DC are distinguished from monocytes and macrophages amongst others by their dendrite morphology, high levels of MHC class II expression, and superior migratory and naïve T-cell priming properties. Transcriptional profiling studies have confirmed that quiescent primary CD1c^+^ cDC, CD141^+^ cDC, and pDC cluster together and form distinct populations separate from monocytes and macrophages (8). However, recent studies have also revealed cases of “mistaken identity” and have challenged several long-standing paradigms regarding the ontogeny and (functional) classification of APC. Especially during inflammation or infection, classifying APC subsets is complicated.

### Monocytes

Monocytes are the most prominent mononuclear phagocyte of the blood compartment and display a wide array of scavenger and pattern recognition receptors that enables them to rapidly respond to danger signals and pathogen encounter. The classical view is that monocytes circulate in the blood for a few days before migrating into tissues where they can develop into macrophages or DC. Renewed interest in recent years has revealed that monocytes may not just be simple transitional and reactive cells but, like DC, display functional diversity and play a critical role in pathogen defense and driving inflammatory diseases.

Human blood monocytes can be distinguished from DC by the expression of CD14, although surface expression levels can vary. It is important to note that while CD11c is a specific DC marker in mice, >95% of human monocytes also express CD11c. Three different types of monocytes have been described based on the expression of CD14 and CD16 (FcγRIII) and classified by expression profiling and hierarchical clustering (4, 22): (1) classical CD14^++^CD16^−^, (2) intermediate CD14^++^CD16^+^, and (3) non-classical CD14^+^CD16^++^ monocytes (Figure 1). A disparity with DC subsets is that monocyte subsets seem to merely represent subsequent developmental stages; although there is no real evidence it is believed that monocytes leave the bone marrow as CD14^++^CD16^−^ and can develop via intermediate CD14^++^CD16^+^ into non-classical CD14^+^CD16^++^ monocytes (22). Classical CD14^++^CD16^−^ monocytes are most prevalent in the blood (about 85% of all monocytes) and are rapidly recruited to tissues in inflammatory responses. CD16-expressing monocytes in general display an advanced stage of differentiation with DC and macrophage-like characteristics and effector functions related to antigen processing and presentation. Numbers of blood CD16^+^ monocytes rise in inflammatory conditions and they represent the main producers of inflammatory cytokines such as TNFα. Especially, the intermediate subtype appears to be highly pro-inflammatory and has the highest capacity to stimulate (antigen-independent) T-cell responses (22, 23). The CD14^dim^CD16^++^ intermediate monocytes can be further subdivided into 6-sulfo LacNAc-positive (slan) and -negative monocytes that both produce high levels of IL-12, TNFα, and IL-1β. The expression of slan was initially identified on an inflammatory human DC subset, the so-called slan DC (24). These cells were found both in blood and skin tissue (24, 25). However, recent data show that slan DC cluster together with, and are indistinguishable from, blood CD16^+^ monocytes (13, 23). This example reveals the difficulties in categorizing APC subsets and even lineages based on surface marker expression and functional characteristics. As will also be discussed further on, CD14^+^ NLT APC, originally classified as DC, may actually represent classical monocytes that have extravasated during steady-state. In line with this, it has been recently challenged whether monocytes become tissue macrophages or DC by default when they exit the bloodstream. Jakubzick and colleagues demonstrated that in steady-state, murine classical monocytes extravasate continuously and carry tissue-derived antigens to the LN without differentiating into macrophages or DC (26). Although monocytes express high levels of MHC class II and also have been shown to be able to retain captured antigen and present antigen-derived peptides, they are relatively poor antigen presenters (21, 23). Alternative mechanisms such as cytokine production or antigen sharing with DC may however contribute to supporting adaptive immunity.

Together, recent data point to a model in which under homeostatic conditions monocytes may not represent “simple” converting cells but play a specific and complementary role to DC in tissue surveillance and promoting adaptive immunity. For full differentiation into tissue macrophages or DC, additional triggers may be required. As will be discussed further on (functional) relationships between monocytes and DC/macrophages are completely different during inflammation.

### Macrophages

Macrophages consist of two types: tissue-resident and infiltrating macrophages. Tissue-resident macrophages are, as opposed to DC and monocytes, long-lived non-migratory cells that are specialized in taking up and processing dead cells and debris. They possess high proteolytic activity and are poor antigen presenters. They play an essential role in maintaining tissue homeostasis by clearing cell debris and promote resolution of inflammation and wound healing (27). However, they can also promote inflammation by the production of chemokines like CCL2, CXCL1, and MIF, and cytokines like IL-6 and TNFα, attracting and activating other immune cells (28, 29).

Tissue macrophages are highly heterogeneous, a consequence of their tissue-specific niches, but functional specialization *in situ* is difficult to establish. The origin of tissue-resident macrophages has long been controversial: are they monocyte-derived or self-maintaining? Recent studies in mice show that under steady-state conditions most tissue macrophages are present from birth and are self-maintaining, independently from monocytes (30–33). As an exception, intestinal macrophages have been shown to be constantly replaced by blood monocytes that in healthy tissue conditions acquire a regulatory expression profile (34). Consistent with the findings of independency from monocytes in mice, several human primary immune deficiencies are associated with severe monocytopenia (35–37) and loss of circulating and tissue DC (35, 36) while tissue macrophages are relatively unaffected. Whether tissue macrophages in humans are completely self-renewing remains to be established since tissue macrophages do not appear to proliferate *in situ* in steady-state (38) and older data also show involvement of BM-derived cells in the replenishment of tissue macrophages following stem cell transplantation (39).

The second type of macrophages, the infiltrating macrophages, is recruited to tissues in inflammatory conditions. In mice, inflammatory monocytes, resembling human classical monocytes and identified by the expression of Ly6c/GR1 and CCR2, have been found to be the source of infiltrating macrophages; distinction between these and resident macrophages can be based on Ly6c expression since Ly6c expression was mostly stable during the first 24 h after infiltration while resident macrophages were negative for this marker (29, 40, 41). In the human setting however, there is still no marker to distinguish infiltrating macrophages from resident macrophages, which makes it hard to study subpopulations. Infiltrating macrophages can be divided into three main populations, with a spectrum of macrophage subpopulations in between, based on function, displaying either a pro-inflammatory profile (originally coined “classically activated” or “M1” macrophages), a regulatory profile, or a wound-healing profile (both originally grouped under the term “alternatively activated” or “M2” macrophages) depending on the tissue context and environmental stimuli (42, 43).

## Functional Specialization Human DC: Steady-State

Due to technical reasons, DC research in humans is largely restricted to peripheral blood-derived DC, using either *ex vivo* primary DC or *in vitro* monocyte-derived DC (Mo-DC). A unique and key feature of DC is their ability to prime naïve T cells. Both cDC subsets, and to a lesser extent pDC, have been shown to prime CD4^+^ T cells, this in contrast to freshly isolated blood monocytes. For efficient *in vitro* CD8^+^ T-cell priming all DC subsets require either TLR stimulation or CD4^+^ T-cell help (20).

### Plasmacytoid DC

Plasmacytoid refers to the non-dendritic, plasma cell-like morphology of pDC in their inactivated state. pDC are characterized by the expression of CD123 (IL-3R), CD303 (BDCA-2), and CD304 (BDCA-4 or Neuropilin-1) and circulate in the blood and LN compartments. Their most notable feature is their ability to quickly secrete large amounts of Type I interferons (IFN) in response to a viral infection (44). pDC selectively express endosomal TLR7 and TLR9 that detect nucleic acids derived from viruses, bacteria, and dead cells. TLR7 or TLR9 ligation triggers a downstream signaling cascade resulting in secretion of IFN-α, IFN-β [reviewed by Ref. (45)], and IFN-λ (46, 47).

In steady-state, pDC have a poor capacity to stimulate CD4^+^ T cells due to their low levels of MHC class II and co-stimulatory receptor expression and limited phagocytosis of antigens. Without stimulation pDC seem to be tolerogenic and are implicated in inducing T-cell anergy and promoting regulatory T-cell development (48, 49). Following stimulation, pDC acquire a dendritic morphology, upregulate HLA-DR and co-stimulatory molecules, and differentiate into functional APC capable of naïve CD4^+^ T-cell activation (45).

### Conventional DC

Both CD1c^+^ and CD141^+^ cDC are found in the blood, LN, spleen, and NLT including skin, liver, lung, and gut. CD1c^+^ cDC co-express CD11b and high levels of CD11c whereas CD141^+^ DC express lower levels of CD11c, lack CD11b, and selectively express CLEC9A (13, 50, 51). The chemokine receptor XCR1 was found to be the most selective marker for both human CD141^+^ DC and mouse CD8α^+^ DC when compared to other DC subsets and monocytes (52, 53). Additionally, the ligand for XCRI, XCL1, selectively attracts CD141^+^ DC and mouse CD8α DC *in vitro* (52, 53) and was shown to be required for optimal *in vivo* priming of CD8 T cells in mice (52).

The two cDC subtypes differ in TLR expression pattern and responsiveness. Whereas CD1c^+^ cDC express all TLR except TLR9, CD141^+^ cDC highly express TLR3 and TLR10, have low expression levels of TLR1-2, TLR6, and TLR8, while they lack TLR4-7 and TLR9 (51, 54). Stimulation of sorted cDC subsets with TLR ligands confirmed restrictive responsiveness of CD141^+^ cDC and showed differential cytokine/chemokine profiles. CD141^+^ cDC were found to predominantly respond to TLR3 ligand (poly I:C) triggering by producing CXCL-10/IP-10, CCL5, and IFN-β. Poly I:C also induced the selective production of IFN-λ, a type III IFN with anti-viral properties (51, 54, 55). Skin-derived CD141^+^ were shown to secrete high amounts of CXCL-10 and TNFα upon TLR3 triggering (13). CD1c^+^ cDC display a broader repertoire of secreted proteins including IL-1β, IL-12, IL-6, TNFα, CXCL8/IL-8, CCL3, CCL4, and CCL5 and CXCL-10 upon TLR3 stimulation (51, 54). Selective high production of IL-12 by CD1c^+^ DC was confirmed upon stimulation with a combination of TLR ligands (20), although use of a different cocktail, of poly I:C plus cytokines, showed that CD141+ DC, too, are well able to produce IL-12p70 (51). Most likely available environmental stimuli will determine local production of this and other cytokines by specific cell subsets.

### Antigen presentation

Primary CD1c^+^ and CD141^+^ have a similar capacity to take up fluorescent protein and they were shown to process and present recombinant protein to autologous CD4^+^ T cells with similar efficiency (51). In addition, they express similar levels of MHC class I and are equally efficient in peptide antigen presentation to autologous CD8^+^ T cells (51). Antigen processing and presentation requires limited degradation of proteins and preservation of cognate T-cell epitopes. Similar to mouse DC, *ex vivo* human pDC and cDC have been shown to contain low proteolytic activity (56). In contrast, Mo-DC show high protein degradation capacity, comparable to that of macrophages (56). With maturation, lysosomal pH drops and degradation capacity increases, which may explain the increased degradation of proteins found in Mo-DC.

### T-cell polarization

Antigen-presenting cells and their secreted products are believed to be decisive in naïve T-cell differentiation and CD4^+^ T_H_-cell subset polarization. Accordingly, CD4^+^ T-cell differentiation was found to be defective in a patient with IRF8 autosomal recessive deficiency, characterized by the lack of monocytes and DC. Whereas *ex vivo* CD4^+^ T-cell proliferation was within normal range, production of IFN-γ, IL-17, and to a lesser extent IL-10, was largely reduced in response to CD3/CD28 or PMA/ionomycin (36).

In an allogeneic MLR both cDC subsets are equally strong stimulators of CD4^+^ T cells regardless of their activation status (51), however T-cell polarization capacities may differ based on the differential TLR and cytokine/chemokine expression profiles. High expression of TLR3 by CD141^+^ DC and their capacity to produce high amounts of IFN-β, CXCL-10, and IL-12p70 all point to T_H_1-inducing function. Indeed, unstimulated CD141^+^ and CD1c^+^ cDC were found to induce T_H_1 differentiation from naïve CD4 T cells, with TLR stimulation further promoting this capacity (51, 54). The highest percentage of IFN-γ-producing cells was observed following TLR3 triggering of CD141^+^ cDC (54). Both unstimulated and poly I:C-stimulated cDC subsets did not induce IL-4, IL-5, and IL-10 production in an allogeneic MLR (51) and, in general, blood DC fail to induce efficient T_H_2 polarization (10). In contrast, LN-resident cDC subsets induce both T_H_1 and T_H_2 cytokine production in naive allogeneic T lymphocytes (10) and skin resident Langerhans cells (LC) have been shown to preferentially induce differentiation of T_H_2 cells (57).

Schlitzer and colleagues have assessed T_H_17 induction by human DC subsets from peripheral blood and lung tissue. Unstimulated lung CD1c^+^ DC were found to express levels of IL-23p19 mRNA higher than those expressed by CD141^+^ DC but comparable to those expressed by CD14^+^ DC/monocytes (12). Consistent with this, both lung and blood CD1c^+^ DC more potently induced IL-17 production by autologous CD4^+^ T cells than CD141^+^ DC and CD14^+^DC/monocytes. Unstimulated CD141^+^ from the liver were however reported to induce both IL-17 and IFN-γ production by allogeneic T cells (58).

### Cross-presentation

Cross-presentation is the presentation of acquired exogenous antigens on MHC class I molecules and is essential for the initiation of CD8^+^ T-cell responses (59, 60). In mice, steady-state LN CD8α DC seem to be the most efficient at cross-presentation (60–62). When CD141^+^ cDC were identified as mouse CD8α^+^ homologs (8) numerous groups subsequently described superior cross-presenting ability of blood CD141^+^ cDC (50–53). Whereas one study showed superior cross-presentation of unstimulated CD141^+^ cDC compared to CD1c^+^ cDC and pDC (53), other groups reported that unstimulated CD141^+^ cDC were unable to cross-present (6, 20, 51). Recent studies have revealed that, with the proper stimuli, all human DC subsets are able to cross-present *in vitro* (63). However, CD141^+^ cDC were shown to be the most efficient cross-presenting subset following poly I:C stimulation (20, 51, 52), when using necrotic cell-associated antigens (51, 53), antigen delivered by Fcγ receptor targeting (64), or antigen delivered to late endosomes/lysosomes (65). The superior ability in cross-presenting necrotic cell-derived antigens may be explained by the selective expression of Clec9A on CD141^+^ cDC (as well as on CD8α^+^ mouse DC), a dead cell receptor that favors cross-presentation through cargo delivery to both MHC class I and II (51).

*In vivo* cross-presentation occurs in secondary lymphoid organs and the Amigorena group has demonstrated that freshly isolated tonsil-resident LN CD1c^+^ cDC, CD141^+^ cDC, and pDC all display equal intrinsic cross-presenting capacity (66). In addition, *ex vivo* unstimulated skin-derived LC and CD141^+^ cells potently cross-present (13, 57). It has therefore been suggested that blood DC may not be fully functional yet and only acquire efficient cross-presenting capabilities after a final step of differentiation in lymphoid organs and tissues (66).

Taken together these data demonstrate that although CD141^+^ have characteristics of specialized cross-presenters compared to other DC subsets, this is highly affected by activation status, location, type of antigen, and inflammatory signals (63). It is also important to note that the requirements for DC subsets to efficiently cross-present may be different than those to stimulate CD8^+^ memory T cells/T-cell clones versus priming of naïve CD8^+^ T cells.

## Functional Specialization Human DC: Inflammation

In an inflammatory setting, monocytes/DC change their phenotype thereby complicating the distinction between phenotypic plasticity within a monocyte/DC population versus discrete subsets. For example, APC can upregulate phenotypic “markers” such as CD14, CD11b, CD141, and CD16, which are used in steady-state to define lineages or functional subsets. Another complicating factor is tissue-instructed differentiation, which confounds hierarchical clustering and principle component analyses. When CD1c and CD141 peripheral blood and tonsilar tissue DC were used for transcriptional profiling and hierarchical clustering, transcriptional activity was more pronounced in tonsil DC and with more transcriptional overlap between the subsets compared to the blood-derived counterparts (7). It was suggested that this is most likely the effect of inflammatory maturation stimuli present in the tonsilar tissue (7) and in general most tissue-specific effects may be due to the induction of different states of maturation. Both mouse and human DC have indeed been shown to undergo profound genetic reprograming *in vivo* during their maturation (67). Interestingly, genes that were identified to be regulated upon DC maturation were irrespective of stimuli, species, or DC subsets. In addition, following an *in vivo* virus challenge mouse DC subsets underwent similar extensive reprograming in their gene expression pattern but maintained their own subset profile, suggesting a common and conserved maturation program without loss of identity (67).

Along with phenotypical changes, functional changes occur following maturation. In general, microbial encounter or inflammatory stimuli change the main function of DC from phagocytosis into efficient priming of T cells. After a transient increase in antigen-uptake, changes in endosomal trafficking and antigen processing lead to increased presentation and, along with upregulation of co-stimulatory molecules, this results in efficient T-cell stimulation. One example of a functional change is the observation that all blood DC can cross-present after stimulation with TLR agonist, while in steady-state the capacity to cross-present is limited (10, 51, 52).

### Inflammatory dendritic cells: The relationship between monocytes and dendritic cells in inflammatory conditions

One of the important outstanding questions in DC biology is the (functional) relationship between DC and monocytes in inflammatory conditions. Monocytes have long been known to be able to differentiate into DC when properly stimulated *in vitro* (68, 69). The best-studied human DC are in fact *in vitro*-generated Mo-DC since they can be generated in large numbers (69). Genome-wide expression profiling has demonstrated that human GM-CSF Mo-DC have more similarities with monocytes than with blood DC subsets (8). Therefore, GM-CSF Mo-DC may share a number of developmental, phenotypic, and functional characteristics with so-called “inflammatory” DC (infDC) – DC derived from monocytes in an inflammatory setting. However, in humans it has never been demonstrated that monocytes give rise to DC *in situ*. In mice, the *in vivo* role of Mo-DC have also long been elusive until in 2003, two studies showed *in vivo* differentiation of monocytes into the above mentioned infDC (70, 71). These infDC were shown to be recruited to the site of inflammation, producing large amounts of TNFα and iNOS (therefore, they were also referred to as TNF–iNOS producing “Tip” DC) and were found to be crucial in pathogen clearance (71). Later, it was demonstrated that infDC are also essential in promoting early pathogen-specific T-cell responses (72), although it has not yet been formally demonstrated whether they are involved in *in vivo* T-cell priming. infDC seem to be specifically important in stimulating T-cell polarization and cytokine production (73). In mice, infDC preferentially induce T_H_1-type responses, but T_H_2 or T_H_17 type responses have also been reported [reviewed by Ref. (73, 74)] depending on the type of (inflammatory) environment. Taken together, monocyte-derived infDC play an important role in the initiation of inflammation and with their rapid and numerous recruitment to inflamed/infected sites and high production of inflammatory cytokines they may act as safeguards that support the function of cDC. Additionally, their highly plastic and versatile nature enables them to respond to local cues and acquire specific activities.

Identifying the *in vivo* human counterpart of infDC has proven to be a challenge. As discussed further on, several infDC phenotypes have been described that are present only in inflamed skin, including Tip-DC and inflammatory dendritic epidermal cells (IDEC). Although all these DC subsets can be considered infDC, their origins and mutual relationship are currently unclear. In the duodenal mucosa of celiac disease patients, there is a rapid accumulation following a gluten challenge of inflammatory CD11c^+^CD14^+^CD163^+^CCR2^+^ DC, which are suggested to be monocyte-derived due to their phenotypic overlap with classical blood monocytes (75). In the lungs of mice, upon inflammatory conditions like respiratory viral infections, an influx of activated inflammatory CD11b and Ly6c-expressing DC is found, together with IFN-producing killer DC that are CD11c^dim^ and B220^+^ [as reviewed in Ref. (76)]. These lung-infiltrating CD11b^+^ Mo-DC were shown to be important in the production of chemokines during allergic inflammation and were capable of inducing T_H_2 cell immunity (74). Additionally, in viral-induced pulmonary inflammation, CD11b^+^ Mo-DC induce strong naïve T-cell proliferation and produce NO synthase 2 (NOS2), playing a predominant role in pathology (77). However, again, how this translates to the human setting remains to be investigated. Another very recent paper describes a specific subset of DC, present in synovial fluid (SF) of RA patients and in inflammatory tumor ascites, that shares molecular features with both conventional CD1c^+^ DC and monocytes/macrophages (78). These CD1c^+^ infDC co-express CD14, CD206, and CD11b, and are enriched for a Mo-DC signature. Although these data indicate a close relationship with monocytes, also here it remains to be established whether they are derived from monocytes or merely represent activated CD1c^+^ DC. In terms of function, CD1c^+^ exudate-derived infDC were demonstrated to promote IFN-γ and IL-17A production by autologous memory T cells and induce IL-17A production by naïve allogeneic T cells and (78) by the production of TGF-β, IL-1β, IL-6, and IL-23, stressing their pro-inflammatory nature. Although exudates are only a partial reflection of what is going on in the inflamed tissues, the highly inflammatory environment and presence of large cell numbers provide an excellent tool to study APC biology and interaction with activated T cells under inflammatory conditions. We have demonstrated that in SF of juvenile idiopathic arthritis patients T cells become resistant to Treg-mediated suppression and that impaired T-cell regulation can be specifically induced by the APC-derived pro-inflammatory cytokines IL-6 and TNFα (79, 80).

### Human DC in chronic autoimmune inflammation

Substantial support is available for the pathogenic role of DC in multiple autoimmune diseases by driving activation and differentiation of effector T-cell populations (81). However, the specific functional roles of DC subsets, as opposed to the general population of DC remains to be established and is one of the future key questions. The best example of the involvement of a specific DC subset in human autoimmune disease so far is the pDC driven type I IFN pathogenesis proposed in several systemic autoimmune diseases. Whereas pDC are largely absent from tissues in steady-state they can accumulate during inflammation. As pDC are not only able to sense viral nucleic acids but also self-nucleic acids in injured tissue leading to their activation and type I IFN production. Tissue infiltration of activated pDC has been reported in skin lesions of SLE, psoriasis, and systemic sclerosis patients (82, 83), salivary glands of Sjögren’s disease patients (84), and muscles and skin of juvenile dermatomyositis patients (85). In these autoimmune diseases, pDC are the major source of type I IFN and are implicated in the initiation of inflammation and the transition to a chronic disease (81).

Migratory tissue DC play a central role in the induction of inflammation. Due to relatively easy accessibility, most human tissue DC work has been performed on skin tissue-derived DC. Therefore, in the next part we will discuss in more detail the function of DC subsets in healthy and (chronically) inflamed skin as a model for human tissue DC biology.

## Skin DC: Steady-State

**Figure 2:**
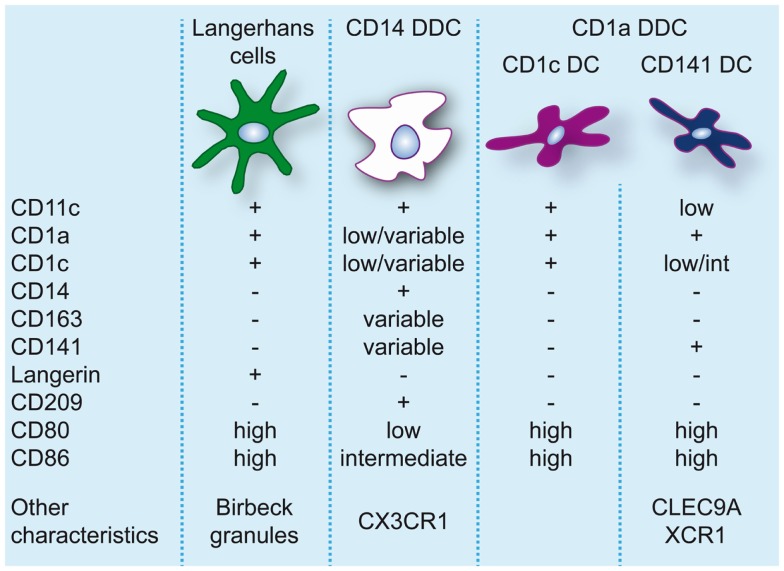
**Dendritic cell populations in human skin during steady-state conditions**. Expression levels of markers commonly used to identify and discern these populations are indicated.

The skin hosts several distinct DC subsets within its two compartments and the classical categorization consists of LC, residing in the epidermis, and CD1a^+^CD14^−^ and CD14^+^ DC, residing in the dermis (86, 87) (for an overview of skin DC in steady-state see Figure 2).

### Langerhans cells

Langerhans cells, due to their location in the epidermis and their protruding dendrites, are a typical example of a DC sentinel, surveying the epidermis for foreign antigens. With their continuous migration to draining LN, they constitute the first immunological barrier of the skin. In human, LC can be identified based on the expression of CD11c, CD32, CD45, FcεR1, high levels of CD1a, CD207/langerin, CD324/E-cadherin, CD326/EpCAM, and HLA-DR, and specific expression of Birbeck granules (88, 89). In steady-state, LC are continuously replaced from a resident precursor pool (90), whereas in inflammation LC are repopulated by blood precursors (91).

*In vitro* human LC are able to take up antigens, process them efficiently and present them to T cells to induce activation, proliferation, and cytokine production (57). They are able to initiate T_H_1, T_H_2, T_H_17, and T_H_22 responses (92). LC migrate to skin-draining lymph nodes in steady-state (10, 93), and LN-derived LC have been found to preferentially induce T_H_2 polarization *in vitro* (10). Additionally, LC are rather efficient at cross-presenting exogenous antigen to naïve CD8^+^ T cells (57, 94).

### Dermal DC

In human dermis, during steady-state conditions, DC are classically divided into CD14^+^ and CD1a^+^ DC (86). CD14^+^ dermal dendritic cells (DDC) show variable expression of CD1a, CD1c, and CD163 (38). Dermal resident macrophages also express CD14 but they can be distinguished from DDC by the lack of CD1c and the presence of high levels of CD163 and FXIIIa and high auto-fluorescence (38, 95). Nevertheless, CD14^+^ DDC express a prominent “mixed” DC/macrophage phenotype and comparative transcriptomic analysis has recently demonstrated the close relation of CD14^+^ DDC with blood monocytes (13, 96), suggesting a monocyte origin. These cells should not be confused with Mo-DC or infDC that only appear during inflammation and display an activated and pro-inflammatory phenotype. CD14^+^ DDC in contrast express low levels of CD80 and CD86 and are relatively poor inducers of naïve T-cell proliferation (38, 57, 95, 97, 98). They do however efficiently take up antigen, possibly due to expression of c-type lectins like CD206 and CD209/DC-SIGN (95, 99). They also have the ability to induce Treg through high production of IL-10 (100).

Skin-derived CD1a^+^ DC express high levels of CD80 and CD86 and strongly induce allogeneic naïve CD4^+^ T cells and CD8^+^ T-cell proliferation (38, 57, 95, 97, 98). CD1a^+^ DDC isolated from skin-draining lymph nodes were found to preferentially induce T_H_2 polarization similar to LC (10). However, human CD1a^+^ DDC seem to be more heterogeneous than originally thought. The majority of CD1a^+^ DDC are uniformly CD1c^high^, but recently CD1c^lo^ DDC highly positive for CD141 were isolated from skin explants. These DC resembled the phenotype of blood CD141^+^ DC, expressing high levels of XCR1, TLR3, CLEC9A, CADM1, and FLT3, and were found to be superior to CD1c^+^ DDC, CD14^+^ DDC, and LC at cross-presenting soluble antigen (13). In contrast to blood CD141^+^ DC cross-presentation by CD141^+^ DDC was even induced in the absence of TLR3 stimulation, which underscores their activated phenotype. Variable expression of CD141 on CD14^+^ DDC has also been reported (13, 100). However, these cells lack the critical features of cross-presenting DC (13) and induce Treg via the production of IL-10 (100).

## Skin DC: Inflammation

**Figure 3:**
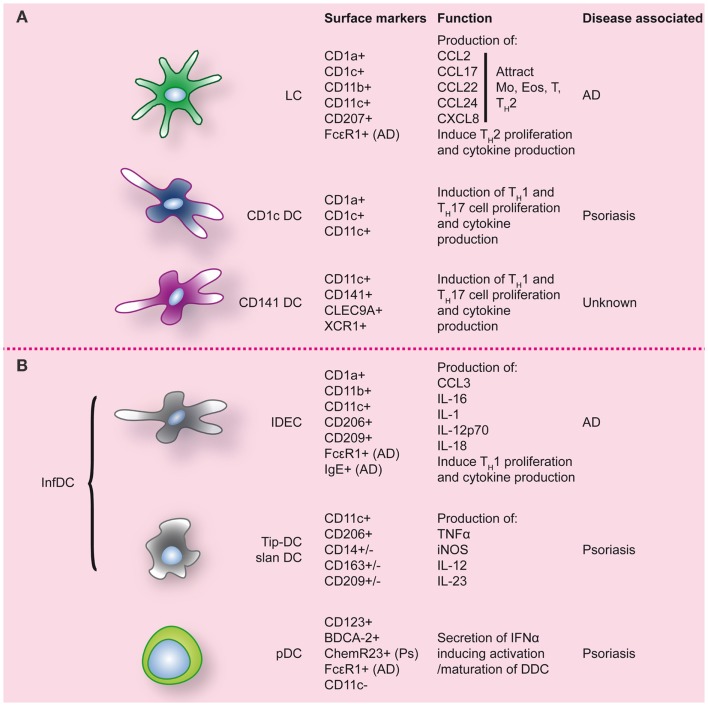
**Dendritic cell populations in human skin during inflammatory conditions**. Inflamed skin contains not only DC that were already present at steady-state **(A)**, but also DC that migrated into the skin **(B)**. Markers commonly used to identify these DC populations are indicated, as well as markers/function associated with specific inflammatory conditions of the skin. LC, Langerhans cell; Ps, psoriasis; AD, atopic dermatitis.

Inflammatory diseases of the skin have been associated with altered DC numbers, suggesting a role of DC in these pathologies. However, what role DC play is often still unclear and remains to be resolved. In both atopic dermatitis (AD) and psoriasis, the two most common inflammatory skin diseases, DC numbers are increased (for an overview see Figure 3). To elucidate the role that DC can play in skin inflammation and inflammation in general, we will draw examples from psoriasis and AD, each with their own T_H_-cell signatures.

### Psoriasis

Psoriasis is a common relapsing immune-mediated inflammatory disease affecting skin and joints, typically presenting as well-demarcated, red, and scaly plaques (101). The typical histological features of psoriatic plaques like thickening of the skin, elongation of the epidermis, and increased numbers and dilated dermal blood vessels, are caused by an immune response gone out of control, in which keratinocytes, DC, and T cells all play a role. In psoriatic lesions, CD8+ T cells are mostly found in the epidermis, while pDC, myeloid DDC and T_H_1 and T_H_17 CD4^+^ cells, are found in the dermis (102).

#### Plasmacytoid DC

In psoriatic lesions, the type I IFN system has been shown to be activated when compared to non-involved skin of the same patient or healthy control (HC) skin (103). Activated pDC are found in increased numbers not only in lesional psoriatic skin (104, 105), but also in uninvolved skin of psoriatic patients, compared to normal skin from HC (104). IFN-α signaling, but not IFN-α expression, is upregulated only in lesional psoriatic skin. Following exposure to light, a well-established treatment for psoriasis, pDC numbers and IFN-α signaling are rapidly reduced in lesional skin, in parallel with a clinical improvement (106). The underlying mechanism of pDC activation in psoriatic skin seems to be via the cathelicidin LL37, an antimicrobial peptide that is produced by keratinocytes and neutrophils and is overexpressed in psoriatic skin. This peptide binds self-DNA/RNA fragments that are released from stressed or dying skin cells, and subsequently triggers TLR7/9 on pDC to induce activation and production of type I IFN (107, 108). Similarly, other proteins secreted by neutrophils via the neutrophil extracellular trap (NET) like HMGB, HNE, CatG, and SLP1, together with DNA induce pDC to produce IFN-α, and possibly play a role in early psoriasis (109–111). Indeed, in a case report, triggering of TLR7 via topical treatment with TLR7 ligand imiquimod was shown to induce exacerbation of psoriasis via massive induction of type I IFN signaling (112). Similarly, in some skin-related cancers, in which imiquimod is used as a topical therapy, imiquimod application strongly increased IFN-α signaling that was related to the number of pDC, suggesting that again pDC-derived IFN-α is responsible for this (113).

While in early and developing psoriasis the role of pDC seems clear, a role in more chronic psoriasis is less distinct. Indeed, when corresponding pDC to phases of psoriasis, pDC are especially found in early and developing psoriasis, and hardly in chronic psoriasis. This temporal dichotomy correlates well with chemerin levels, a chemokine that attracts chemR23-expressing pDC and is highly expressed in pre-psoriatic skin and early lesions (114, 115).

#### Dermal DC

While pDC are considered to play a vital role in the development of psoriatic lesions, DDC are thought to be of major importance in the more chronic phase of psoriasis. In psoriatic dermis, CD11c^+^ DC are increased up to 10 times compared to non-lesional skin (98, 116, 117). T cells surrounding the DDC were found to be persistently activated and differentiated toward a type 1 effector phenotype, both cytotoxic and T_H_ cells (118, 119). Psoriatic lesion-derived DDC were found to be more potent inducers of spontaneous proliferation of autologous T cells, especially of the T_H_1 subtype producing high amounts of IFN-γ and IL-2, than DDC from HC skin or cDC from peripheral blood of psoriatic patients (98, 118). Others show that psoriatic skin-derived DDC not only induce T_H_1 but also T_H_17 polarization (120). DDC from psoriatic patients are indeed a potent source of IL-23 (117, 121–123) and IL-12, although literature remains in discord on whether or not there is an increase of IL-12 expression in psoriatic skin compared to normal skin (122, 124, 125).

Dermal dendritic cells in psoriatic skin can be further subdivided into three subsets: CD1c^+^ DC, CD141^+^ DC, and CD11c^+^CD1c^−^CD141^−^“inflammatory” DC. Compared to normal skin, CD1c^+^ DC numbers are decreased and CD141^+^ DC increased in both non-lesional and lesional skin of psoriatic patients. However, the increase in infDC [up to 30 times compared to normal skin (120)] is largely responsible for the total increase of CD11c^+^ cells in psoriatic lesional skin. Both CD1c^+^ and CD1c^−^ DDC populations from psoriatic skin are able to induce T-cell proliferation and production of IFN-γ and/or IL-17 to the same extent (120).

#### Inflammatory DC

“Inflammatory” CD11c^+^CD1c^−^CD141^−^DDC, expressing high levels of TNF-α and iNOS, specifically accumulate in psoriatic lesions (126). In analogy with the mouse DC subset, they have been termed Tip-DC (126). Dermal Tip-DC express some CD14, CD163, and CD209/DC-SIGN (120) and are assumed to be monocyte-derived. The pathogenic impact of these DC is thought to result from the production of pro-inflammatory cytokines. TNFα induces keratinocytes to express ICAM-1, CXCL8, and also pro-inflammatory cytokines like IL-1β and IL-6 (102). iNOS in inflamed tissues catalyzes the production of nitric oxide (NO) that in inflamed skin can lead to vasodilation of dermal blood vessels in psoriatic skin (102). In addition Tip-DC have been shown to produce high levels of IL-23, a pro-inflammatory cytokine driving Th17 polarization and strongly associated with psoriasis (127, 128). Following effective therapy in psoriasis, TNFα, iNOS, and IL-23 production by Tip-DC is strongly reduced. A (sub-)population of DC in psoriasis was demonstrated to express 6-SulfoLacNac and display similarities with peripheral “slanDC” (25). The complete transcriptional overlap of blood slanDC with CD16^+^ monocytes, also suggests a monocyte origin of skin slanDC (13, 23). Like pDC, dermal slanDC are reactive to self-RNA-LL37 complexes (25) and their high IL-1β, IL-6, TNFα, IL-12, and IL-23 production results in T_H_1/T_H_17 T-cell programing (25). Because of their phenotypic signature, it is likely that dermal Tip-DC and slanDC represent the same infDC population although subpopulations may exist. In conclusion, accumulating dermal infDC seem to play a key role in the progression of psoriasis and sustenance of psoriatic lesions by secreting large amounts of pro-inflammatory mediators including iNOS, IL-12/IL-23, and TNFα. The latter cytokines, and especially IL-23, have been proven effective targets for clinical therapy in psoriasis.

#### Langerhans cells

A vital role for LC in psoriasis has not been described thus far. Compared to normal skin, LC frequency and phenotype are normal in uninvolved skin of psoriatic patients. However, mobilization of LC toward lymph nodes in response to allergens or cytokines like TNFα and IL-1β, factors that normally induce migration of these cells, was largely absent (129). Furthermore, culture of psoriatic patient-derived monocytes into LC (mLC) showed migratory capacity similar to those cultured from HC-derived monocytes, suggesting that there is no intrinsic DC defect but an underlying altered epidermal microenvironment in psoriasis patients (130). Such local change may not only influence LC function, which can be used to distinguish between early-onset and late-onset psoriasis (131), but perhaps also be the basis for the development of psoriasis.

Together, there seems to be a pivotal role for DC in the pathogenesis of psoriasis, not only during the initiation, but also the sustenance of the disease. However, especially in the non-acute phase of the disease, it remains difficult to determine what the exact role is of each DC subset. Moreover, further definition of these subsets needs to be addressed. While CD11c^+^ CD1c^−^ inflammatory DDC were presented as a separate DC subset (71), gene expression profiling of CD11c^+^CD1c^+^ and CD11c^+^CD1c^−^ DDC shows that these two populations are closely related (132), suggesting that the inflammatory DDC are perhaps a more activated form of the CD11c^+^CD1c^+^ DDC instead of a distinct subset.

### Atopic dermatitis

Atopic dermatitis, or atopic eczema, like psoriasis, is a common relapsing inflammatory disease affecting the skin. AD patients typically present with eczematous patches and plaques, histologically defined by epidermal intercellular edema (spongiosis) and prominent dermal cellular infiltration. In AD skin, T-cell numbers are increased, consisting of mostly T_H_2 cells in the acute phase while there is a prominent T_H_1 aspect in the subacute chronic phase (133–135). Additionally, like in psoriasis, skin DC numbers are increased in general, particularly DC expressing CD11c^+^ and CD1a^+^ (136).

#### Plasmacytoid dendritic cells

In contrast to psoriasis, there seems to be no role for pDC in AD pathogenesis, since they are virtually absent from AD lesions like in healthy skin (105). However, peripheral pDC in AD carry the FcεRI that, when activated induces IL-10 production, while IFN-α production is dramatically decreased compared to that by pDC from HC (137). Together, this may explain the increased susceptibility of AD patients to viral infections.

#### Langerhans cells and inflammatory epidermal dendritic cells

Two subsets of FcεRI-bearing DC are most important in AD. Firstly, LC that show a slightly changed phenotype compared to steady-state (89). Additionally, infiltrating IDEC are present (89). Since the majority of these cells are found in the dermis of AD (136), they are also referred to as myeloid inflammatory dendritic cells (MIDC).

Both LC and IDEC express high levels of FcεRI (89, 138, 139). The expression of this FcεRI, in combination with a low expression of CD32/FCγRII, strongly distinguishes AD patients from patients with other lesional skin conditions like psoriasis and allergic dermatitis (140). During the acute phase of AD, in which lesions are characterized by a T_H_2 signature, LC may capture antigens via FcεRI-bound IgE and become activated, leading to secretion of CCL2/MCP-1 and IL-16, and possibly CXCL8 (141). This induced attraction of monocytes, eosinophils, and T cells. Additionally, LC can internalize, process, and present IgE-associated allergens to T cells, which can take place locally or in skin-draining LN, resulting in a polarization toward T_H_2 cells and production of T_H_2 cytokines like IL-4, IL-5, IL-13 (141). LC also express high levels of the receptor for TSLP, a ligand that is secreted in ample amounts by keratinocytes. Binding TSLP leads to LC maturation, survival, and secretion of CXCL8, CCL17, CCL22, CCL24, and IL-15, driving T_H_2 recruitment. TSLP-induced LC activate T_H_2 cells, thereby enhancing the T_H_2 profile in AD (141, 142).

In the subacute and chronic phase of AD, lesions are more characterized by a T_H_1 signature. After migration into the skin monocytes are thought to differentiate into IDEC, the DC subtype that seems to be responsible for the chronic maintenance phase of AD. Phenotypical analysis shows that IDEC are CD1a^+^, CD1b^dim^, CD1c^dim^, CD11b^bright^, CD11c^+^, CD23^dim^ CD32^dim^, CD36^bright^, CD206^bright^, CD209^+^, FcεRI^bright^, IgE^+^, HLA-DR^bright^, and negative for lineage markers (89, 136, 140, 143). After epicutaneous exposure of AD patients to aero-antigens and food antigens, in most cases an eczematous reaction ensued, in which case FcεR1-bearing IDEC were found in the epidermis within 72 h (144). Upon ligation of FcεRI, these IDEC are able to produce CCL3, IL-1, IL-16, but also IL-12p70 and IL-18 (141). These latter two cytokines induce T_H_1 polarization and production of IFNγ, thus likely contributing to the switch from T_H_2 to T_H_1 that leads to the chronic phase of the disease (133).

Together, also for AD, there seems to be a key role for DC in the pathogenesis of the disease. In this case, the main role during genesis of the disease is assigned to LC, while pDC hardly play a role. For the chronic phase again there is an infDC type, but one different from the infDC type prominent in psoriasis. Interestingly, although skin of AD and psoriasis patients harbors DC subsets with different characteristics, *ex vivo* DC subsets from both disease groups show similar T-cell polarizing ability (145). In contrast, chemokine expression differs markedly between the two diseases, showing T_H_2-associated and some T_H_1-associated chemokines in lesional AD skin, while demonstrating mostly T_H_1/T_H_17-associated chemokines in psoriasis (145). These data would suggest that skin DC do not directly drive T-cell polarization but contribute indirectly by shaping the local chemokine environment and attracting and further stimulating specific subsets of T cells that are recruited to in the skin. It is important to realize however that DC responses largely depend on the nature and extend of the stimulus. While *ex vivo* triggered DC subsets may show a high degree of functional plasticity, the tissue context and local inflammatory environment likely polarize and restrict their *in vivo* function.

## Conclusion

Over the past years, there has been important progress in our understanding of human DC subsets and their functional roles in steady-state conditions. With transcriptional profiling data distinct subsets of circulating and tissue DC have been defined and their relationships with monocytes and macrophages have been further elucidated. In inflammatory conditions, plasticity, and versatility of the DC and monocyte compartments is revealed with local signals driving their differentiation and function. The challenge ahead is to define the contributions of DC subsets in these (pathogenic) inflammatory conditions and its resolution.

## Author Contributions

Both Arjan Boltjes and Femke van Wijk: conception and design, drafting, and revising. Both authors approve of the final version to be published and agree to be accountable for all aspects of the work.

## Conflict of Interest Statement

The authors declare that the research was conducted in the absence of any commercial or financial relationships that could be construed as a potential conflict of interest. The Guest Associate Editor Marianne Boes declares that, despite being affiliated to the same institution as authors Arjan Boltjes and Femke van Wijk, the review process was handled objectively and no conflict of interest exists.
